# Antimicrobial Resistance in Typhoidal Salmonella: Surveillance for Enteric Fever in Asia Project, 2016–2019

**DOI:** 10.1093/cid/ciaa1323

**Published:** 2020-12-01

**Authors:** Farah N Qamar, Mohammad T Yousafzai, Irum F Dehraj, Sadia Shakoor, Seema Irfan, Aneeta Hotwani, Muhammad J Hunzai, Rozina S Thobani, Najeeb Rahman, Junaid Mehmood, Caitlin Hemlock, Ashraf M Memon, Jason R Andrews, Stephen P Luby, Denise O Garrett, Ashley T Longley, Kashmira Date, Samir K Saha

**Affiliations:** 1 Department of Pediatrics and Child Health, Aga Khan University, Karachi, Pakistan; 2 Department of Pathology and Laboratory Medicine, Aga Khan University, Karachi, Pakistan; 3 Applied Epidemiology, Sabin Vaccine Institute, Washington, DC, USA; 4 Kharadar General Hospital, Karachi, Pakistan; 5 Infectious Diseases and Geographic Medicine, Stanford University, Stanford, California, USA; 6 National Foundation for the Centers for Disease Control and Prevention, Atlanta, Georgia, USA; 7 Global Immunization Division, Centers for Disease Control and Prevention, Atlanta, Georgia, USA; 8 Child Health Research Foundation, Department of Microbiology, Dhaka Shishu (Children) Hospital, Bangladesh

**Keywords:** enteric fever, antimicrobial resistance, multidrug resistance, extensive drug resistance, Asia

## Abstract

**Background:**

Clinicians have limited therapeutic options for enteric as a result of increasing antimicrobial resistance, and therefore typhoid vaccination is recommended as a preventive measure. As a part of the Surveillance for Enteric Fever in Asia Project (SEAP), we investigated the extent measured the burden of antimicrobial resistance (AMR) among confirmed enteric fever cases in Bangladesh, Nepal, and Pakistan.

**Methods:**

From September 2016–September 2019, SEAP recruited study participants of all age groups from its outpatient, inpatient, hospital laboratory, laboratory network, and surgical sites who had a diagnosis of febrile illness that was either suspected or blood culture confirmed for enteric fever. Antimicrobial resistance of isolates was determined by disc diffusion using Clinical and Laboratory Standard Institute cut-off points. We reported the frequency of multidrug resistance (MDR)(resistance to ampicillin, cotrimoxazole, and chloramphenicol), extensive drug resistance (XDR) (MDR plus non-susceptible to fluoroquinolone and any 3rd generation cephalosporins), and fluoroquinolone (FQ) and azithromycin non-susceptibility.

**Results:**

We enrolled 8,705 blood culture confirmed enteric fever cases: 4,873 (56%) from Bangladesh, 1,602 (18%) from Nepal and 2,230 (26%) from Pakistan. Of these, 7,591 (87%) were *Salmonella* Typhi and 1114 (13%) were *S*. Paratyphi. MDR *S*. Typhi was identified in 17% (701/4065) of isolates in Bangladesh, and 1% (19/1342) in Nepal. In Pakistan, 16 % (331/2084) of *S.* Typhi isolates were MDR, and 64% (1319/2074) were XDR. FQ nonsusceptibility among *S.* Typhi isolates was 98% in Bangladesh, 87% in Nepal, and 95% in Pakistan. Azithromycin non-susceptibility was detected in 77 (2%) in Bangladesh, 9 (.67%) in Nepal and 9 (.59%) isolates in Pakistan. In Pakistan, three (2%) *S.* Paratyphi isolates were MDR; no MDR *S*. Paratyphi was reported from Bangladesh or Nepal.

**Conclusions:**

Although AMR against *S*. Paratyphi was low across the three countries, there was widespread drug resistance among S. Typhi, including FQ non-susceptibility and the emergence of XDR *S*. Typhi in Pakistan, limiting treatment options. As typhoid conjugate vaccine (TCV) is rolled out, surveillance should continue to monitor changes in AMR to inform policies and to monitor drug resistance in S. Paratyphi, for which there is no vaccine.

Enteric fever, caused by *Salmonella enterica serovar* Typhi (*S*. Typhi) and *Salmonella enterica serovar* Paratyphi (*S*. Paratyphi) remains a major public health problem in many parts of Asia and Africa, commonly occurring in pediatric populations [1–3]. It is an acute systemic infectious disease that was responsible for an estimated 14.3 million cases of typhoid and paratyphoid fever and 135.9 thousand deaths globally in 2017 [4]. Multidrug resistance (MDR) and fluoroquinolone (FQ) non-susceptibility are increasingly reported for *S.* Typhi and *S.* Paratyphi in South Asia and Africa [5–8].

Surveillance studies demonstrate considerable geographic variation in the proportion of *S*. Typhi isolates that are MDR within the same region, with sites in Nepal, India, Pakistan, and Vietnam having higher rates of MDR than sites in China and Indonesia [1, 9]. A declining trend of MDR typhoid in South Asia has been reported, with the exception of Pakistan, where drug resistance continues to be high [10]. In Bangladesh, a decline in the isolation rate of MDR strains was reported between 2004–2016 [11], with a similar observation being reported from India and Nepal [12–16]. Some suggest that with a significant decrease in MDR strains, cheaper and more effective first-line antibiotics may re-emerge as drugs of choice for the treatment of typhoid fever in these countries [12], although this idea needs to be evaluated in clinical practice before any change in treatment recommendations are proposed. Outbreaks of MDR strains in Nepal and Bangladesh have led to the widespread use of FQ as a first line therapy [17]. However, over 90% of *S*. Typhi isolates have been non-susceptible to fluoroquinolones in most Asian countries, including Bangladesh, Nepal, and Pakistan for the last several years [6, 18–20]. Growing non-susceptibility to antibiotics, especially ceftriaxone and FQ, severely limits the treatment options.

The emergence of extensively drug resistant (XDR) *S*. Typhi is an added threat in the face of ongoing high rates of non-susceptibility to other antimicrobials. Drug- non-susceptibility is not only associated with severity of illness, but it also increases the length of stay in the hospital and the cost of treatment, and it can result in higher morbidity and mortality [21]. The situation is particularly worrisome in resource-limited settings where the burden of disease is high and the few remaining effective antimicrobials are either unavailable or too expensive for public health services or the general public [22]. Diagnosis of typhoid, in endemic countries, as shown in a recent report from Nepal, depends on physicians’ clinical judgment [23]. The probability of sending a blood culture for diagnosis depends on multiple factors: duration of fever, presence or absence of signs and symptoms of other diseases, and prior antibiotic use. These factors, coupled with limited availability of facilities for blood culture, leads to underreporting of the true number of cases of blood culture-confirmed typhoid [24, 25].

While enteric fever is endemic in South Asia, reliable population-based antimicrobial resistance surveillance data are lacking. The Surveillance for Enteric Fever in Asia Project (SEAP) was a large, multi-center, prospective surveillance study capturing data on the burden of enteric fever and the antimicrobial susceptibility of the isolates. From 2016 to 2019, SEAP used a uniform methodology to enroll patients with culture-confirmed enteric fever at participating sites in Bangladesh, Nepal, and Pakistan, to record laboratory data, clinical metadata and patient outcomes.

The objective of this study was to measure the burden of antimicrobial resistance (AMR) in *S.* Typhi and *S.* Paratyphi isolates among patients with enteric fever in Bangladesh, Nepal, and Pakistan. The results from this study will contribute important data on AMR for comparison across and within these countries. Additionally, the data can be used as an important baseline measurement of AMR before the introduction of typhoid conjugate vaccine (TCV) into routine immunization programs in these three countries.

## METHODS

### Study Design, Sites and Participants

This prospective surveillance study was conducted at hospitals and laboratory networks in Bangladesh, Nepal and Pakistan. The study recruited cases from Dhaka Shishu (Children’s) Hospital and Shishu Sasthya (Pediatric) Foundation Hospital in Bangladesh; Dhulikhel Hospital and Kathmandu Medical College and Teaching Hospital in Nepal; and the Aga Khan University Hospital (AKUH), Kharadar General Hospital (KGH), Jinnah Postgraduate Medical Center (JPMC) and the National Institute of Child Health (NICH) in Pakistan. Cases were also recruited from the laboratory networks, including the Popular Diagnostic Centers (Dhanmondi, Mirpur, and Shamoly branches), Bangladesh; the Alka Hospital, Nepal Medical College, Kathmandu Model Hospital, Bir Hospital, Helping Hands Clinic, Nepal Police Hospital, Kanti Children Hospital, and Nepal Army Hospital, Nepal; and the Aga Khan University Laboratory Network and AKUH main laboratory collection unit, Pakistan.

### Case Definition and Enrollment Criteria

Study participants included individuals of all age groups at outpatient, inpatient, hospital laboratory, laboratory network and surgical sites, with a diagnosis of febrile illness that was either suspected or blood culture-confirmed for enteric fever. Outpatients from a defined catchment area [26] with fever for ≥3 days in the last 7 days and advised blood culture were enrolled into the study. For inpatients, all suspected or culture-confirmed cases were enrolled. Patients from the hospital laboratories and laboratory network sites were identified from lab reports; if their blood culture was positive for *S*. Typhi or *S*. Paratyphi they were contacted by telephone and enrolled in the study.

### Laboratory Samples and Analysis

A trained phlebotomist at designated SEAP sites collected a blood sample immediately after the participant was enrolled and before antibiotic administration. Samples were incubated overnight at 37°C. Blood culture bottles were processed on BD Bactec automated blood culture system. Gram stain and subsequent subcultures were performed on Sheep Blood Agar, MacConkey’s Agar, and Chocolate Agar from samples positive by Bactec. Positive cultures suggestive of *S*. Paratyphi and *S*. Typhi were confirmed serologically with BD Difco™ Salmonella O Antiserum Factor 9, Antiserum Vi, and Antiserum Factor 2 antisera. All isolates were tested for their susceptibility as per Clinical and Laboratory Standard Institute Guidelines-M100-ED-29, 2019. Isolates were multidrug resistant (MDR) if they were resistant to ampicillin/amoxicillin, chloramphenicol, and trimethoprim-sulfamethoxazole. Isolates were fluoroquinolone (FQ) non-susceptible if they had intermediate susceptibility or were non-susceptible to ciprofloxacin and were extensively drug resistant (XDR) if they were MDR and were also non-susceptible to fluoroquinolone and any 3^rd^ generation cephalosporins.

### Data Collection and Analysis

Trained research associates with a nursing or medical background interviewed the patients or their caretakers to collect socio-demographic and other information related to the illness. We reviewed medical charts and files to gather data on clinical and laboratory testing and diagnosis of any complications. Data were entered on tablets using electronic case report forms. Descriptive analyses such as AMR by age, gender, recruitment areas, countries and months were performed. Complications such as hepatitis, hemodynamic shock, pulmonary complications, gastrointestinal complications and sepsis were compared between MDR/XDR *S*. Typhi and non-MDR/XDR typhoid patients using Fisher’s Exact test [18].

### Ethical Considerations

Written informed consent was obtained from adults (18+ years) and parents or legal guardians for children; assent along with parental consent was taken for children 15 to 17 years old. Participants could withdraw consent at any time. A unique identifier, with no personal identifying details, was assigned to maintain confidentiality. All consent/assent forms, signatures, and personal details were kept in locked cabinets accessible by the project supervisor and principal investigator of the respective country. All tablets and computers were password protected with a secured central server system to archive data. The study was approved by the Bangladesh Institute of Child Health Ethical Review Committee, Nepal Health Research Council Ethical Review Board Approval, Ethical Review Committee of Aga Khan University and National Bioethics Committee of Pakistan, and Stanford University. This project was approved by the Centers for Disease Control and Prevention’s human subjects review as “research with CDC not directly engaged,” so a full IRB approval was not needed.

## RESULTS

A total of 8,705 culture confirmed enteric fever cases were enrolled from the SEAP sites in all three countries: 4,873 (56%) from Bangladesh, 1,602 (18%) from Nepal, and 2,230 (26%) from Pakistan. Of these, 7,591 (87%) were *S*. Typhi and 1, 114 (13%) were *S*. Paratyphi. (Table 1). Among the 1,095 *S.* Paratyphi isolates for which drug resistance testing results were available, only 3 (.3%), all from Pakistan, were MDR (Table 1). Among the 7,491 *S.* Typhi isolates that had susceptibility testing, 1,051 (14%) were MDR: 17% of isolates in Bangladesh, 1% of isolates in Nepal, and 16% in Pakistan. Among 7,098 *S.* Typhi isolates tested, 1,319 (19%) were XDR. All XDR isolates were from Pakistan; 64% of all *S*. Typhi isolates in Pakistan were XDR (Table 1).

**Table 1.** Socio-demographic and Clinical Characteristics of Culture - Confirmed Enteric Fever Cases - Bangladesh, Nepal and Pakistan, September 2016–September 2019

	Bangladesh				Nepal				Pakistan				Total			
Countries	*S.* Typhi		*S.* Paratyphi		*S.* Typhi		*S.* Paratyphi		*S.* Typhi		*S.* Paratyphi		*S.* Typhi		*S.* Paratyphi	
	n = 4131	%	n = 742	%	n = 1367	%	n = 235	%	n = 2093	%	n = 137	%	n = 7591	%	n = 1114	%
Age in Ys <2 y	394	9.54	55	7.41	15	1.10	3	1.28	299	14.29	6	4.38	708	9.33	64	5.75
≥2–5 y	1146	27.74	168	22.64	39	2.85	7	2.98	609	29.10	13	9.49	1794	23.63	188	16.88
≥5–15 y	1666	40.33	272	36.66	279	20.41	34	14.47	784	37.46	34	24.82	2729	35.95	340	30.52
≥15–25	564	13.65	124	16.71	707	51.72	111	47.23	242	11.56	46	33.58	1513	19.93	281	25.22
≥25	361	8.74	123	16.58	327	23.92	80	34.04	159	7.60	38	27.74	847	11.16	241	21.63
Sex																
Male	2330	56.40	424	57.14	793	58.01	149	63.40	1197	57.19	89	64.96	4320	56.91	662	59.43
Recruitment Location																
Inpatient	660	15.98	72	9.70	31	2.27	7	2.98	724	34.59	24	17.52	1415	18.64	103	9.25
Outpatient	1269	30.72	184	24.80	218	15.95	48	20.43	629	30.05	65	47.45	2116	27.88	297	26.66
Hospital Laboratory & Lab Network	2202	53.30	486	65.50	1117	81.71	180	76.60	732	34.97	48	35.04	4051	53.37	714	64.09
Surgery	0	0	0	0	1	.07	0	0	8	0	0	0	9	.12	0	0
MDR^a^																
Yes	701/4065	17.24	0/735	0	19/1342	1.42	0/226	0	331/2084	15.88	3/134	2.24	1051/7491	14.03	3/1095	.27
No	3364/4065	82.76	735/735	100	1323/1342	98.58	226/226	100	1753/2084	84.12	131/134	97.76	6440/7491	85.97	1092/1095	99.73
Not performed	66		7		25		9		9		3		100		19	
XDR^b^																
Yes	0/4064	0	0/735	0	0/940	0	0/166	0	1319/2074	64.0	0/132	0	1319/7078	18.64	0/1033	0
No	4064/4064	100	735/735	100	940/940	100	166/166	100	755/2074	36.0	132/132	100	5759/7078	81.36	1033/1033	100
Not performed^c^	67		7		427		69		19		5		513		81	

^a^Multidrug Resistance (MDR): Resistance to ampicillin/amoxicillin, chloramphenicol, and trimethoprim-sulphamethoxazole.

^b^Extensively drug Resistance (XDR): MDR with fluoroquinolone and any 3rd generation cephalosporin resistance.

^c^Not performed: at least one of the antibiotics were not tested in the respective group of antibiotics that make it MDR or XDR).

Resistance of *S.* Typhi isolates to first-line antibiotics (ampicillin, cotrimoxazole, and chloramphenicol) varied by country, ranging from 18%–27% in Bangladesh, 2%–3% in Nepal, and 82% in Pakistan (Table 2). Ceftriaxone resistance was found in 65% of *S.* Typhi isolates in Pakistan, but none among isolates in Bangladesh or Nepal.

**Table 2.** Distribution of Antimicrobial Resistance Among Culture Confirmed Enteric Fever Patients - Bangladesh, Nepal and Pakistan, September 2016–September 2019

	Bangladesh	Nepal	Pakistan	Total
Countries	n = 4131	n = 1367	n = 2093	n = 7591
*S.* Typhi	%	%	%	%
Ampicillin				
Resistant	27.31	2.97	82.50	38.33
Sensitive	72.69	97.03	17.50	61.67
Cotrimoxazole				
Resistant	18.11	2.15	82.08	33.04
Sensitive	81.89	97.85	17.92	66.96
Chloramphenicol				
Resistant	18.84	1.93	81.50	33.25
Sensitive	81.16	98.07	18.50	66.75
Ceftriaxone				
Resistant	.00	.21	65.25	19.22
Sensitive	100.00	99.79	34.75	80.78
Ciprofloxacin				
Resistant/Intermediate	97.74	86.77	95.06	95.02
Sensitive	2.26	13.23	4.94	4.98
Azithromycin				
Resistant	1.89	.67	.59	1.37
Sensitive	98.11	99.33	99.41	98.63

FQ non-susceptibility in *S*. Typhi was very high in all three countries (98% in Bangladesh, 87% in Nepal, and 96% in Pakistan), and non-susceptibility to azithromycin was detected in 77 (2%) isolates in Bangladesh, 9 (.66%) in Nepal and 9 (.58%) in Pakistan. (Table 2, Figure 2).

**Figure 1.**
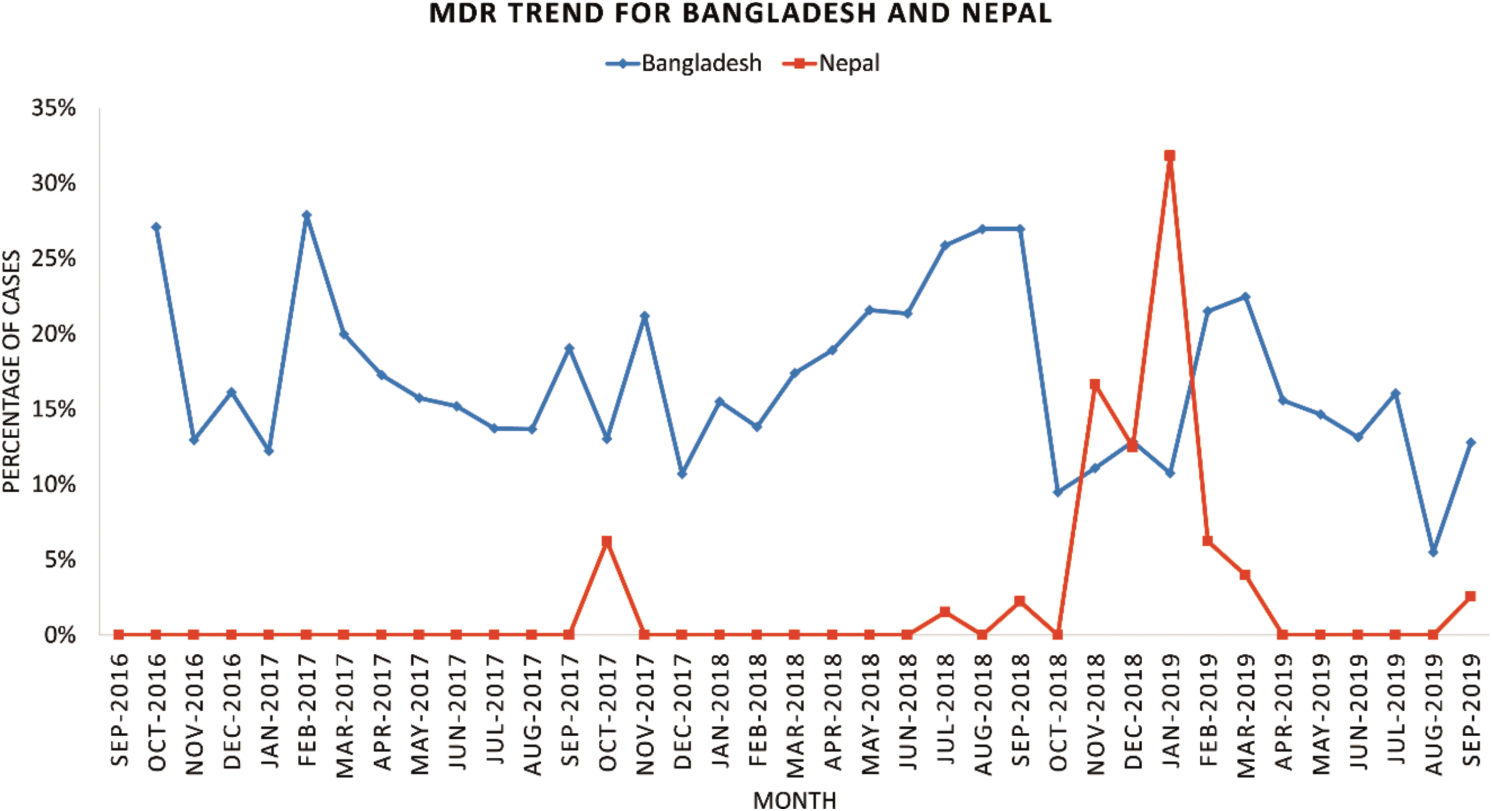
Trend in proportion of *S*. Typhi isolates with multidrug resistance - Bangladesh and Nepal, September 2016–September 2019. Abbreviation: MDR, multidrug resistance.

Trends in *S*. Typhi resistance over time varied by country. The proportion of enteric fever cases with MDR isolates was relatively stable over time in Bangladesh (about 15%–20% of all isolates per month), with fewer MDR isolates found in late 2018 and toward the end of the surveillance period in 2019 (Figure 1). In Nepal, there were almost no MDR isolates except for a 5-month period during November 2018–March 2019. (Figure 1).

**Figure 2.**
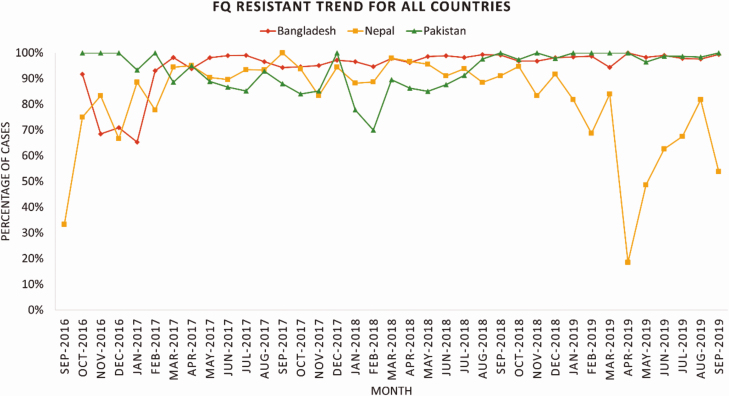
Trend in proportion of *S*. Typhi isolates with fluoroquinolone non-susceptibility - Bangladesh, Nepal and Pakistan, September 2016–September 2019, Bangladesh, Nepal, and Pakistan. Abbreviation: FQ, fluoroquinolone.

FQ non-susceptibility among the patients with *S.* Typhi was generally uniform in all three countries during the study period except for a decrease in Nepal during November 2018–April 2019 (Figure 2).

**Figure 3.**
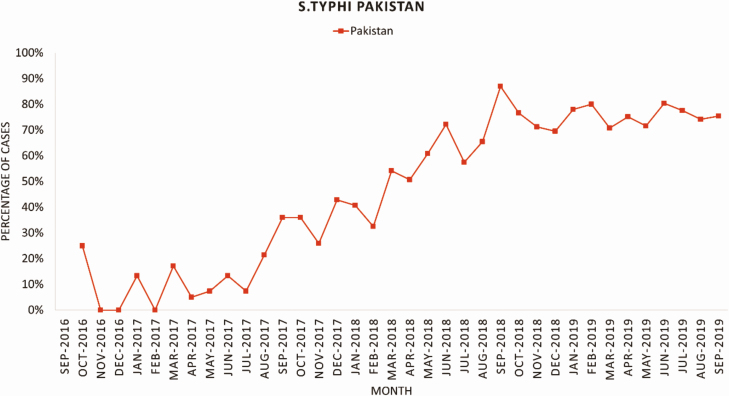
Time trend of proportion of *S*. Typhi isolates with extensive-drug resistance September 2016 - September 2019, Pakistan.

In Pakistan, there was an increasing trend of XDR isolates among the patients with *S*. Typhi during 2016–2019 (Figure 3). Complications of severe enteric fever, such as hepatitis, hemodynamic shock, pulmonary complications, gastrointestinal complications, intestinal perforation and sepsis occurred more frequently among patients with XDR/MDR *S*. Typhi compared with non-XDR/MDR *S*. Typhi cases (Table 3). However, the proportion of complications reported were infrequent in both groups. Only the occurrence of one or more complications was higher in the MDR/XDR typhoid group and statistically significant when compared with S. Typhi non-MDR/ XDR cases.

**Table 3.** Complications Between Drug Resistant and Sensitive Cases of S. Typhi in Karachi, Pakistan, September 2016–September 2019

	*S*. Typhi (MDR/XDR Cases)		*S*. Typhi (non MDR/ XDR Cases)		
Complications	n = 1650	%	n = 443	%	*P*value
Hepatitis	20	1.2	1	.2	.1
Hemodynamic shock	13	.8	0	0	.083
Pulmonary complications	10	.6	0	0	.13
GI complication	9	.6	0	0	.22
Intestinal perforation	8	.5	0	0	.22
Sepsis	6	.4	0	0	.35
Intestinal obstruction	6	.4	1	.2	1
Encephalopathy	3	.2	2	.5	.29
Renal impairment	2	.1	0	0	1
Blood cell disorders	2	.1	0	0	1
Others (myocarditis/wound infection/disseminated intravascular coagulation (DIC) /musculoskeletal complications/electrolyte imbalance)	4	.2	0	0	.58
At least one or more than one complications iIndividuals)	58	3.5	4	.90	.005
All Admissions^a^	n = 710 mean (SD)		n = 145 mean (SD)		
Duration/Days of hospitalization	9.98 (7.87)		10.43 (10.61)		.555
Temperature at admission^b^	101.45 (1.63)		101.18 (1.89)		.109

^a^All admissions includes patients recruited at OPD, IPD, Hospital Lab, Surgical.

^b^Temperature at admission: *S*. Typhi (MDR/XDR Cases) n = 687/710, information not available n = 23. *S.* Typhi (non MDR/ XDR Cases) n = 144/145, information not available n = 1.

*S.* Typhi = 831/855, information not available n = 24.

## DISCUSSION

This study reported the antimicrobial resistance pattern from tertiary care hospitals and laboratory networks from three endemic countries, Bangladesh, Nepal, and Pakistan. Surveillance for antimicrobial resistance among enteric fever isolates from SEAP project revealed high rates of antimicrobial non-susceptibility among *S*. Typhi isolates to FQ in all three countries and high rates of XDR *S*. Typhi in Pakistan. Among patients with *S*. Paratyphi, MDR was currently low and only seen in Pakistan. Given the paucity of available antimicrobials, the potentially emerging azithromycin non-susceptibility in these three countries is a threat for typhoid treatment in endemic countries.

MDR in *S.* Typhi has increased, while in *S.* Paratyphi it has decreased markedly [27]. Previous data from Pakistan suggest the prevalence of MDR *S.* Typhi increased from 20% in 1992 to approximately 50% in 2015, whereas the MDR in *S*. Paratyphi has declined since 2004 to almost negligible levels. In Pakistan, most MDR *S.* Typhi have acquired additional resistance to beta-lactams and FQ, making them extensively drug resistant [18]. In contrast with Pakistan, where high rates of XDR are reported, the proportion of MDR in Bangladesh and Nepal was low [21, 28].

The epidemics of MDR typhoid in several countries in the 1980s and 1990s were the driving force for making ciprofloxacin the drug of choice for the treatment of typhoid [22, 24, 25]. Fluoroquinolone non-susceptibility in *S*. Typhi started rising in 2002 and reached 96.5% globally in 2015 [15, 27]. Fluoroquinolones are not only extensively prescribed for humans but are widespread in animal husbandry. Sale of fluoroquinolones is generally unregulated in developing countries [29–31]. In low and middle-income countries, patients may not have access to or are prevented from attending health facilities due to high out of pocket costs; therefore, they seek treatment over the counter from community pharmacies or in the informal sector [32]. In general, a direct relationship has been found between the amount of antibiotic used and the frequency of non-susceptibility to multiple antibiotics [33]. The progressive appearance of non-susceptibility against fluoroquinolone made third-generation cephalosporins (ceftriaxone and cefixime) the treatment of choice for typhoid in endemic countries [34, 35]. Now, with the emergence of XDR typhoid in Pakistan, treatment options for typhoid are limited to azithromycin and carbapenems [18].

Azithromycin is the only oral antibiotic remaining for treatment for patients with XDR typhoid. The potential emergence of azithromycin non-susceptibility is an added threat; if acquired by the XDR strain of *S.* Typhi, typhoid may become virtually untreatable in outpatients. If antibiotics continue to be used as compensation for poor water and sanitation and weak health systems, the battle against typhoid will be lost [33]. The emergence of non-susceptibility among the commonly used antibiotics for typhoid has been associated with a worse clinical response, higher rates of complications and deaths, as well as prolonged fecal shedding, which sustains transmission and propagates secondary cases within communities [36–38].

In Asia, and more recently in Africa, children with MDR typhoid have been reported to be sicker and more toxic at admission compared to patients with *S*. Typhi sensitive to antimicrobials. Drug resistant typhoid leads to high rates of complications, prolonged hospital stays, increased treatment costs, and subsequent higher risk of mortality [39, 40]. Longley et al paper in this supplement.

One way of addressing the issue of antimicrobial resistance may be through the use of TCV [41]. In Bangladesh and Pakistan, the high number of cases aged <15 years has implications for policy decisions for a typhoid vaccination strategy. Yousafzai et al paper in this supplement. The choice of routine immunization at 9 months versus a onetime catch-up campaign in children 9 months–15 years, followed by routine introduction, would vary depending on the disease burden, disease severity (hospitalizations and deaths), treatment, and intervention costs in these countries [42]. In the presence of high disease burden in children with high rates of AMR, a onetime catch-up campaign followed by routine introduction might be a cost-effective vaccination strategy in these settings [17, 43].

The rise in AMR in enteric fever in high disease burden countries, particularly the increase in XDR typhoid, is also a global security concern. Reported cases of imported XDR typhoid among travelers from Pakistan highlight the need for coordinated global actions for effective surveillance, improvements in water and sanitation, and implementation of effective vaccines [44]. TCV has the potential to be an effective tool to fight against all *S*. Typhi, irrespective of AMR status [45].

Better diagnostics for typhoid are needed, since, for every one true case of typhoid fever, 3 to 25 patients without typhoid are treated with antimicrobials leading to overuse and misuse of antimicrobials [46]. The paucity of microbiology facilities and the consequent lack of antimicrobial susceptibility testing data to inform empirical antimicrobial treatment have ramifications beyond enteric fever. There is a need for low cost, sensitive diagnostics for typhoid and paratyphoid that can be made widely available to facilitate in typhoid management and control. Blood culture, which is the existing “gold standard” has limitations of sensitivity, costs, trainings and quality control [47]. This study included blood culture surveillance, but many enteric fever endemic areas lack routine blood culture surveillance in the absence of special studies.

This study had several strengths. The use of a standard case definition and the identification of culture-confirmed enteric fever cases, limited misclassification and linked clinical data to AMR data. The data for this study were collected prospectively over three years, hence the seasonal trends inferred are likely reliable. Limitations of the study included collection of complications data from medical charts, which could have underestimated the scope. Additionally, we were not able to characterize the azithromycin isolates by genotyping to assess if there were any differences in molecular patterns of non-susceptibility by site from the three countries. Furthermore, though there is no comparative evaluation of blood culture sensitivity between drug sensitive and MDR enteric fever infections to indicate a reduced sensitivity of blood cultures in MDR or XDR typhoid, one study on quantitation of *S*. Typhi in blood in a Vietnamese cohort indicated that bacterial counts were higher in patients with drug resistant typhoid fever [48]. It is therefore possible that AMR might have affected the sensitivity of blood culture in the SEAP study population.

## CONCLUSION

The rise in FQ non-susceptibility, the emergence of XDR *S*. Typhi, and the potential for azithromycin non-susceptibility is a warning sign for the global community to expedite efforts for the control of enteric fever. The results from this study provide an important database on the antimicrobial resistance patterns among *S.* Typhi isolates in Bangladesh, Nepal, and Pakistan, for comparison across and within these three countries. Additionally, the data can be used as an important baseline measurement of AMR before the introduction of TCV in routine immunization programs in these three countries. Although the potential for identifying new drugs for enteric fever is not encouraging, the routine surveillance of AMR is still critical to assess the effectiveness of antimicrobial regimens and to guide local and national treatment policies.

## Notes

Notes

***Financial support.*** This work was supported by the Bill & Melinda Gates Foundation (grant number INV-008335). Agreement (sub-contract/sub-grant) between the Sabin Vaccine Institute (Primary grantee for the Surveillance for Enteric Fever in Asia Project grant from the Bill and Melinda Gates Foundation) and the CDC Foundation.

***Supplement sponsorship***. This supplement is sponsored by the Sabin Vaccine Institute and made possible by a grant from the Bill & Melinda Gates Foundation.

***Potential conflicts of interest.*** S. K. S. reports grants from Child Health Research Foundation. All other authors report no potential conflicts. All authors have submitted the ICJME form for disclosure of potential conflict of interest. Conflicts that the editors consider relevant to the content of the manuscript have been disclosed.
